# Probing the Cultural Constitution of Causal Cognition – A Research Program

**DOI:** 10.3389/fpsyg.2016.00245

**Published:** 2016-02-23

**Authors:** Andrea Bender, Sieghard Beller

**Affiliations:** Department of Psychosocial Science, Faculty of Psychology, University of BergenBergen, Norway

**Keywords:** causal cognition, culture, language, methods, interdisciplinary approach

## Abstract

To what extent is the way people perceive, represent, and reason about causal relationships dependent on culture? While there have been sporadic attempts to explore this question, a systematic investigation is still lacking. Here, we propose that human causal cognition is not only superficially affected by cultural background, but that it is co-constituted by the cultural nature of the human species. To this end, we take stock of on-going research, with a particular focus on the methodological approaches taken: cross-species comparisons, archeological accounts, developmental studies, cross-cultural, and cross-linguistic experiments, as well as in-depth within-culture analyses of cognitive concepts, processes, and changes over time. We argue that only a combination of these approaches will allow us to integrate different components of cognition, levels of analysis, and points of view—the key requirements for a comprehensive, interdisciplinary research program to advance this field.

## Introduction

Causal cognition refers to how people perceive, represent, and reason about causal relationships, that is, about the connection between a causing event and its effects. Grasping such relationships is essential to people’s daily lives—for example when trying to understand the laws of nature, social interactions with other people, or causes and treatments of illness—and hence arguably a core concern of human cognition. But does this render cognitive engagement with causality a universal and uniform phenomenon? While decades of research into causal cognition have helped to unravel important principles on which causal perception, learning, or reasoning are based (for an overview, see Waldmann and Hagmayer, 2013), the extent to which these processes and their outcomes may depend on culture has received substantially less attention (for one prominent line of research, see Norenzayan and Nisbett, 2000).

Here, we defend the position that people’s cultural background *does* affect whether and how they engage in causal cognition: by shaping the settings in which causal cognition occurs, the manner in which potential factors are pondered on, the selection of relevant factors from a causal field, or the way in which such factors are linguistically expressed. Although on-going research already provides some supporting evidence for this position, efforts need to be intensified to substantiate it. As this poses methodological challenges, we place a particular focus on the various approaches taken, in order to outline a research program for the investigation of cultural impacts in this domain more generally.

Research approaches to cultural influences are necessarily pre-structured by whatever definition of “culture” one chooses. While we have no intention of perpetuating a centuries-old debate that cannot, in principle, be resolved, we do consider it important to outline what the topic does and does not include. Taking an anthropological perspective (Bender and Beller, 2011b) and following Tylor’s (1871, p. 1) classic definition, we understand “culture” as including the knowledge, beliefs, capabilities, and habits that people have acquired as members of society (for a recent debate on the term *culture*, see Brumann, 1999, and the comments to it). As this operationalization of culture encompasses language, not only as a medium but also as a core component, evidence of linguistic influences on causal cognition will also be treated as relevant instances.

Based on this working definition, we first briefly describe different methodological approaches taken to investigate the question at stake, before explicating why we consider it fundamental to combine these approaches in order to obtain a comprehensive perspective on the cultural influences on causal cognition.

## Methodological Approaches—An Overview

Causal cognition in a more narrow sense has been a key topic in philosophy and cognitive psychology, and has therefore mainly been tackled through conceptual analyses and psychological experiments. The repertoire of possible approaches, however, is substantially larger, ranging from ethnographic observations to statistical analyses of linguistic data, thereby spanning a wide spectrum of disciplines. It includes cross-species comparisons, archeological accounts, developmental studies, cross-cultural and cross-linguistic experiments, as well as in-depth within-culture analyses of cognitive concepts, processes, and changes over time.

### Cross-Species Comparisons

Even if we are actually interested in those aspects of human causal cognition that may be affected by culture and are thus variable, an understanding of what is shared by humans seems indispensable. One question to be asked is therefore: Which components of causal cognition are invariant across species, and which are distinct to humans and set them apart from their closest phylogenetic relatives, the great apes (Hanus et al., 2011), or other highly intelligent and social species like crows (Taylor, 2014) or rats (Blaisdell et al., 2006)? Answers to this question would not only provide a baseline for further investigations, but a valuable window into the complex interactions between changes in causal cognition brought about by individual learning, cultural transmission, and evolution.

As causal cognition in non-human species cannot be directly investigated, tool use is typically taken as an indicator of causal understanding (overview in Taylor and Gray, 2014). Comparative research on human and non-human causal cognition has not yet reached generally agreed-upon conclusions. Penn and Povinelli (2007), for instance, concede in their critical review that non-human causal cognition appears to be significantly more sophisticated than traditional association theories of causal learning would allow for, but they also claim that non-human species are probably not capable of any human-like causal-logical inferences about abstract causal relations. This conclusion is contested now by experimental studies which demonstrate that some of the great apes base their behavior on inferences from causal cues and perhaps even on a deeper understanding of causal principles (Mendes et al., 2007; Hanus and Call, 2011), and some corvids even take hidden causal agents into account (Taylor et al., 2012; and see Taylor, 2014).

Despite the controversy in this field, comparative research across species remains indispensable for delineating those components of causal cognition that are specific to humans, and thus potentially susceptible to cultural impact, from those that are part of our phylogenetic heritage. These attempts need to take into account, however, that not only humans but also many non-human species form complex societies, which are partly based on learning and hence are able to pass on behaviors culturally, at least to some extent (for debate on this, see, e.g., Laland and Hoppitt, 2003; Whiten and van Schaik, 2007).

### Archeological Accounts

Once we know which components of causal cognition are specifically human, we can begin to ask questions such as: did they emerge as a by-product of cognitive evolution, or were they acquired during individual learning, stored and scaffolded by cultural means? Questions like these lie in the scope of paleontology and archeology; yet attempts to trace the evolution of human causal cognition through archeological records have commenced only very recently (Haidle, 2014). Due to the sparseness of available data, such studies have also remained largely theoretical, focusing on tool use as indicator of causal reasoning and understanding in an even more indirect way than the comparative psychological research described above (McCormack et al., 2011; Taylor and Gray, 2014).

The key idea is to consider the construction of tools (the material remains of which constitute virtually the only available data on past human cognition and behavior) as attempts to increase a person’s agency by solving a concrete problem. Typically, the process of constructing and optimizing tools for specific purposes presupposes an understanding of, or at least experimentations on, cause-effect relations. The distance between the perceived problem and the contrived solution has increased continuously over the course of human evolution: from the use of simple tools, through effective chaining for secondary and modular tools, to composite tools, complementary tool sets, and the highly abstract use of notional tools based on symbols (Haidle, 2014).

Although this archeological analysis, functioning as a kind of reverse engineering, only provides us with rough estimates of the time points in human evolution when certain components of causal cognition must have been in place, it nevertheless helps us to specify not only the changes that took place, but also the processes by which they were presumably brought about, for example, in terms of culturally relevant ontologies that underpin and influence causal reasoning processes (Alberti and Bray, 2009). Another example that might also provide archeological evidence for causal reasoning is the origin and sophistication of farming techniques, combining tool use with natural studies and social factors (Mithen, 2007).

### Developmental Studies

An alternative approach disentangling the complex interactions between individual learning, cultural transmission, and evolutionary influences on causal cognition is taken in developmental psychology, where the assumption is widespread that a child’s responses, observed in infancy, or unfolding at fixed ages as a product of maturation, can be taken as evidence for concepts and cognitive processes deeply rooted in phylogeny and ontology.

Spelke and Kinzler (2007), in particular, conceptualize such “core knowledge” as distinct domain-specific modules for reasoning about physical, biological, and social or psychological events. Each domain is defined by entities which have specific causal properties, marked, for example, by the way they move: Physical entities are set in motion by external forces, while animates, the inhabitants of the biological world, can move on their own initiative. The psychological/social domain, finally, is populated by sentient agents, whose behavior is caused by mental states such as knowledge, beliefs, goals, and intentions.

Such a priori knowledge is investigated with children who are as young and unaffected by culture as possible, taking looking time, and gaze direction as indicators (e.g., Sobel and Kirkham, 2006; Rakison and Krogh, 2012; for a critical position see Saxe and Carey, 2006). Although these studies do provide valuable data on components of causal cognition that may be universal, respective inferences would be much stronger if they were not tested almost exclusively in children from a Western urban background. Drawing on broader samples not only allows for more reliable generalizations, but may also change the very inferences which are reached. For instance, not all generalizations drawn from the core-knowledge approach hold cross-culturally, as shown for the biological domain in a series of studies by Medin and colleagues (e.g., Medin and Atran, 2004; Bang et al., 2007; Medin et al., 2014; Ojalehto et al., 2015).

### Cross-Cultural and Cross-Linguistic Experiments

The gold standard for scrutinizing cultural influences has been cross-cultural comparisons of participants’ assessments of causal scenarios. One way of doing this, which aims at tapping into perception and attribution tendencies, is by collecting verbally expressed assessments of animated displays or vignettes that visualize or describe causal events. While such studies are typically alike in that they yield cultural differences, for instance in which of the involved entities is assigned more causal relevance, the inferences the researchers draw regarding the underlying mechanisms diverge substantially. Morris and Peng (1994), for instance, attribute the differences to implicit theories, which favor a general focus on either internal or external causes of behavior (Peng and Knowles, 2003), whereas others point at linguistic cues and content variables as the relevant factors for causal assignments (Beller et al., 2009; Bender and Beller, 2011a; and see Le Guen et al., 2015).

As languages differ substantially in how they encode information about causal relations and events (e.g., Wolff et al., 2009; Bohnemeyer et al., 2010), cross-linguistic studies and linguistic variations can help to uncover the role that language may play in shaping causal cognition—not only as a medium of culture, but also as a factor in its own right. Two sets of studies on this question indicate that a change of the linguistic framing can be sufficient to shift people’s attention to different aspects of an event (e.g., from the causer to the affected object), with consequences for memory processes, the assignment of blame, and even the severity of punishment for human agents involved (Beller et al., 2009; Fausey and Boroditsky, 2010; Fausey et al., 2010).

Higher-order cognitive and behavioral differences based on distinct causal understanding and representations have also been tackled in cross-cultural studies (e.g., Güss et al., 2010), but require more sophisticated tasks and designs. One approach taken in research on complex problem solving, for instance, is to construct microworlds in which participants are responsible for retaining a balance between several interconnected factors (overview in Güss and Robinson, 2014). These studies yield data sets that tend to be richer but also more complex than simple causal assignment tasks.

### In-Depth Within-Culture Analyses

If the underlying theoretical models are sufficiently concrete to afford experimental designs rather than the quasi-experimental designs of cross-cultural research, or if the ethnographic information is sufficiently detailed to warrant in-depth analyses, it is also possible to test predictions and interpretations within cultures and languages. In an early study of this type, the anthropologist Kempton (1986) showed how different types of thermostat usage can be traced back to different cultural models of how these artifacts function. More recently, studies employing experimental designs uncovered that different cultural models of causal dependencies may co-exist in the same mind and are accessed in different contexts (Astuti and Harris, 2008; Legare and Gelman, 2008; and see Tucker et al., 2015). Likewise, linguist Alessandro Duranti (1994) demonstrated how linguistic cues such as ergative marking of agency may be used in diverse contexts to emphasize or deemphasize responsibility. And also the extent to which a property like intentionality is considered when judging wrong-doing may depend on context (Astuti and Bloch, 2015; Sousa et al., 2015).

A particularly detailed account of how respective information can be gleaned from ethnographic descriptions of cultural contexts that no longer exist—and critical remarks on the diligence needed to assess them—is provided by Widlok (2014). He emphasizes that culture-specific notions of time, linearity, and sequence as well as extensions of agency and personhood are crucial for a more thorough understanding of cultural differences in causal cognition. The account contains a caveat on the malleability of “culture” over time and depending on context and perspective. This point is also emphasized by Iliev and Ojalehto (2015), who discuss within-culture diachronic analyses as a necessary complement to cross-cultural investigations. Specifically, they describe automated text analysis as a promising tool to investigate how attention to, and notions of, causality may have changed over time within a cultural/linguistic context, thus shedding more light on the complex links between culture, language and cognition.

## The Need for and Benefits of Combining Different Approaches

The studies described in the previous section take radically different methodological approaches to investigate the cultural constitution of causal cognition, and their findings are not, prima facie, easy to integrate. We still argue that combining these approaches, and the insights they yield, is indispensable for obtaining a comprehensive understanding of the question at stake—for at least three reasons: They allow us to target different *components of cognition*, to take different *levels of analysis* into account, and to broaden our perspective by considering the *points of view* of other disciplines.

### Components of Cognition

The methodological approaches described above target partly different components of cognition. While, for instance, comparative research across species, and archeological recapitulations of human evolution are both concerned with tool use as core indicator of causal understanding in problem-solving (Taylor and Gray, 2014), they still differ in their specific interests, with the former focusing more on the cues assumed to facilitate causal perception and learning (e.g., Hanus et al., 2011), and the latter more on forward-planning and the organization of problem-solving processes (Haidle, 2014).

More generally, the components tackled by any of these approaches include perceptions and representation of causal relations (e.g., Bender and Beller, 2011a; Le Guen et al., 2015; Ojalehto et al., 2015; Tucker et al., 2015), learning processes and outcomes (Blaisdell et al., 2006), verbal expressions and explanations (Iliev and Ojalehto, 2015), as well as higher-order cognitive effects on attention, memorization, and recall (Fausey et al., 2010), categorization and inferences (Medin and Atran, 2004), causal attributions in social interactions (Morris and Peng, 1994), judgments of blameworthiness and punishment (Fausey and Boroditsky, 2010; Astuti and Bloch, 2015), problem-solving in complex situations (Güss and Robinson, 2014), strategies on how to handle artifacts in daily life (Kempton, 1986), and ways in which illnesses are explained and treated (Hagmayer and Engelmann, 2014; Luhrmann et al., 2015).

Combining the different approaches and their insights therefore yields a much more comprehensive understanding not only of what causal cognition may encompass, but also of the various ways in which culture may actually influence it.

### Levels of Analysis

As the various methodological approaches target different components of cognition, they also allow different levels of analysis to be taken into account. These levels range from a long-term, evolutionary level (*phylogenesis*), through changes over historic time (*sociogenesis*), to small-scale development in infants and children (*ontogenesis*), and to actual cognition (*microgenesis*).

These different levels of analysis can be fruitfully combined to complement and refine each other. For instance, both the statistical corpus-based analysis of verbal material by Iliev and Ojalehto (2015) and the detailed conceptual analysis of old ethnographic material by Widlok (2014) rely heavily on text, while taking divergent approaches: one that grossly abstracts over the topic and details of texts, and one that sees these very factors as essential for getting to the gist. The former (Iliev and Ojalehto, 2015) highlights the extent to which the concern with a specific topic, the usage of respective vocabulary, and perhaps even the conceptual ingredients can change over (historic) time within a given population. The detailed conceptual analysis by Widlok (2014), in turn, pinpoints how much contextual information is actually needed to glean valuable information from concrete descriptions (see also Astuti and Bloch, 2015). Combining them could therefore help to further improve them both: by giving more precision to the statistical analysis on the one hand, and by broadening the context for the ethnographic exploration on the other.

### Points of View

In line with the levels of analysis that the different methodological approaches target, these approaches are also characteristic for specific disciplinary traditions, including archeology, paleontology, primatology, and anthropology, various subfields of psychology (like comparative, developmental, cognitive, and social psychology), computational linguistics, (ethno-)linguistics, and philosophy. Half a century of efforts to establish an integrative cognitive science may have taught us that this is a difficult endeavor (e.g., Bender et al., 2010; Beller et al., 2012)—but it is one that is worth the trouble.

Engaging in successful cross-disciplinary research activities requires a great deal of reflexivity among the scholars involved, and both *interactional expertise* and *epistemic awareness.* The former is the linguistic ability to engage *about* the practices of another discipline without the ability to actually engage *in* these practices (Collins and Evans, 2002), while the latter is a metacognitive understanding that different research practices carry with them assumptions about what constitutes ‘good science’ which need to be respected to enable fruitful critical dialog between the participating researchers (Nersessian, 2015).

Combining various perspectives across disciplines is not only a value in and of itself, but also promises greater insights (Barrett et al., 2012; Bender et al., 2015) and even novel research questions (e.g., Osbeck et al., 2011; Luhrmann et al., 2015; Moya et al., 2015) that would eventually help to advance the intrinsically interdisciplinary field of causal cognition. Exemplary cases of such bridge-building work include, among others, the collaborations of anthropologists and psychologists on the co-existence of cultural models regarding causal dependencies (Astuti and Harris, 2008; Watson-Jones et al., 2015), on causal reasoning in the biological domain (e.g., Medin and Atran, 2004; Bang et al., 2007), and on explanations for disorders (Hagmayer and Engelmann, 2014; Luhrmann et al., 2015).

## Conclusion

Humans are inherently social and cultural beings. Their very success as a species over time hinged on the fact that cultural transmission dramatically propelled the learning process (Tennie et al., 2009; Tomasello, 2014). How this distinctively human power, in turn, impacts on the way humans perceive, represent, and reason about causal relationships is still poorly explored. It is thus high time for an interdisciplinary multi-level research program that investigates the cultural constitution of causal cognition more systematically for its various components, on different levels of analysis, and from diverse points of views.

## Author Contributions

This article represents joint work of both authors.

## Conflict of Interest Statement

The authors declare that the research was conducted in the absence of any commercial or financial relationships that could be construed as a potential conflict of interest.
